# Erythrocyte Nitric Oxide at High Altitude in Mountaineers Susceptible to High-Altitude Pulmonary Edema

**DOI:** 10.1016/j.jacadv.2023.100720

**Published:** 2023-11-22

**Authors:** Pascal B. Meyre, Stefanie Zuegel, Stephanie Kiencke, Christoph Dehnert, Beat A. Kaufmann

High-altitude pulmonary edema (HAPE) is characterized by hypoxic pulmonary vasoconstriction with subsequent pulmonary hypertension and increased capillary pressure after rapid ascent to high altitude.^1^ Imbalance in vasoconstriction/vasodilatation and reduced nitric oxide (NO) production might play a key role in the development of HAPE. Erythrocytes carry NO bound to thiol (transnitrosylated hemoglobin, SNO:Hb) or bound to heme (Fe:NO) and are able to release biologically active NO in a hypoxic environment. Levels of NO:Hb inversely correlate to pulmonary artery pressure in patients with pulmonary hypertension.^2^ We aimed to investigate changes in erythrocyte NO metabolism at high altitude between HAPE susceptibles (HAPE-S) and those resistant to HAPE (HAPE-R) and examine whether defects in erythrocyte NO metabolism play a role in the increase in pulmonary artery pressure at high altitude.

Mountaineers with a history of HAPE (HAPE-S) and HAPE-R were prospectively studied with echocardiograms and blood samples at low altitude (490 m) and after rapid ascent to high altitude (4,559 m). Participants were excluded if they had a recent stay at high altitude within the last 30 days (>5 nights at altitude >2,500 m). Written informed consent was obtained from all participants, and the study was approved by the Ethical Committee of the University of Zurich.

Participants were transported to Alagna, Italy (1,205 m), ascended by cable car to 2,900 m, hiked to a hut at 3,647 m for an overnight stay, and then climbed to 4,559 m the next morning. Measurements at high altitude were taken the following morning after an overnight stay (minimum 24 hours). Systolic pulmonary artery pressure was assessed through transthoracic echocardiography at both low and high altitudes, followed by arterial and venous blood sampling at each altitude. Blood gases were measured immediately. For measurement of arterial and venous erythrocyte NO:Hb molar ratios (as total [NO:Hb], bound to thiol [SNO:Hb] or to heme [FeNO:Hb]), blood was immediately transferred into a nitrogen-filled glove box. Erythrocytes were separated from plasma by centrifugation, resuspended in a cryopreservation solution, and snap frozen immediately in liquid nitrogen. The samples were always kept at −80 °C until analysis. Values were compared between HAPE-S and HAPE-R using t-test for normally distributed or Wilcoxon rank-sum test for non-normally distributed continuous variables. A repeated measures analysis of variance with Tukey post hoc test was used for multiple comparisons in data with a normal distribution, and a Kruskal-Wallis test with Dunn’s post hoc test was used for multiple comparisons in non-normally distributed data.

**Figure 1:**
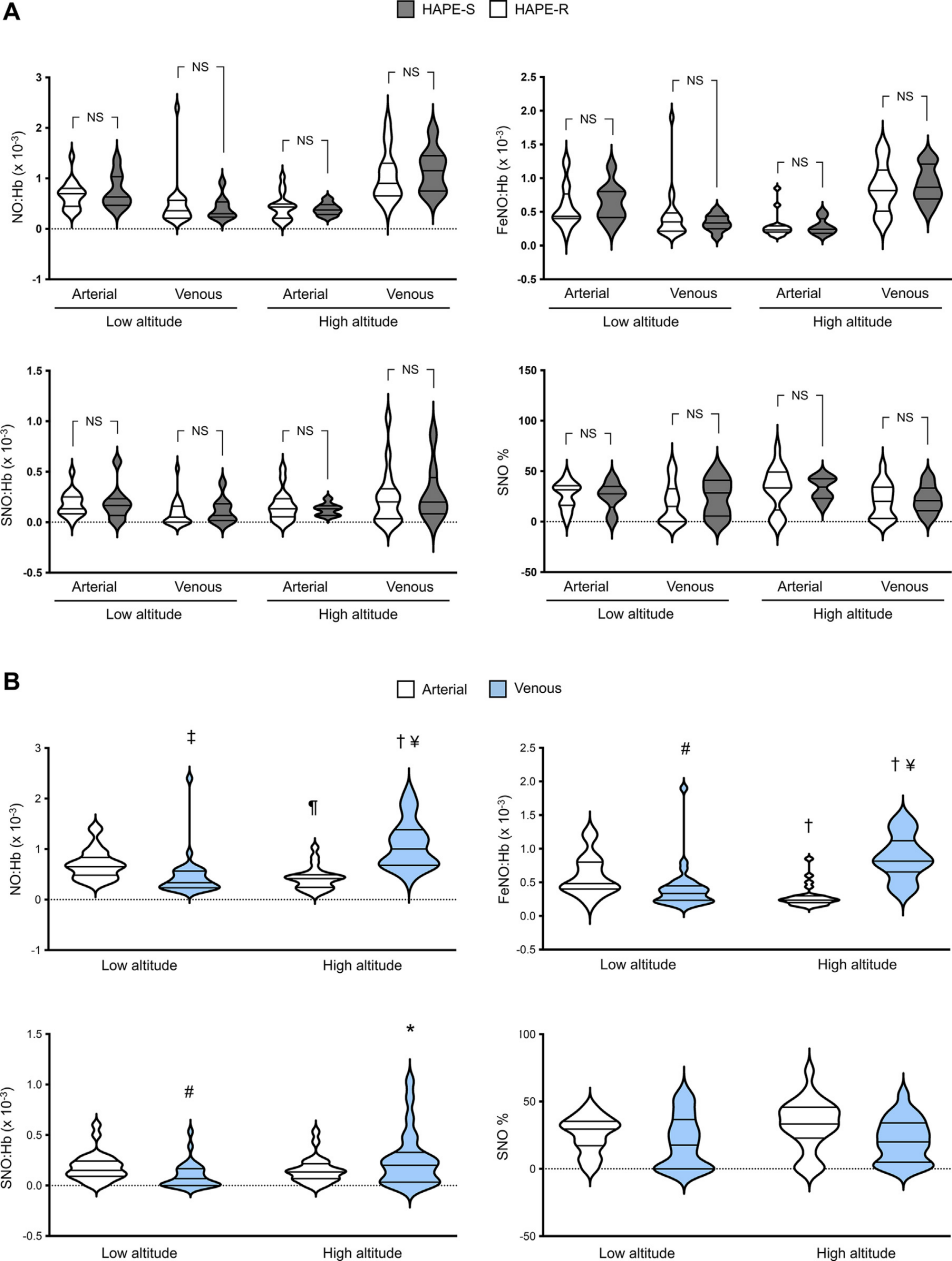
Erythrocyte NO Content (A) Arterial and venous NO:Hb, SNO:Hb, FeNO:Hb, and SNO% in HAPE-resistant and HAPE-susceptible subjects at low and high altitude. (B) Pooled analysis for NO:Hb, FeNO:Hb, SNO:Hb, and SNO% at low and high altitude. HAPE-R = HAPE-resistant; HAPE-S = HAPE-susceptible; NS = nonsignificant. ∗*P* < 0.05 for high vs low altitude; ¶*P* < 0.01 for high vs low altitude; †*P* < 0.001 for high vs low altitude; #*P* < 0.05 for venous vs arterial blood; ‡*P* < 0.01 for venous vs arterial blood; ¥*P* < 0.01 for venous vs arterial blood.

A total of 24 mountaineers (8 with and 16 without a history of HAPE) were analyzed. Mean age was 44 ± 9 years, and 40% were female. At high altitude, hemoglobin oxygen saturation decreased by 15 to 20%, and the sPAP increased from 20 ± 3 mm Hg to 38 ± 8 mm Hg in HAPE-R and from 21 ± 3 mm Hg to 47 ± 9 mm Hg in HAPE-S participants (*P* < 0.05). The molar ratios of NO:Hb, SNO:Hb, and FeNO:Hb between HAPE-R and HAPE-S in arterial and venous blood samples are shown in Figure 1A. There were no significant differences between the 2 groups. In pooled analyses including all mountaineers, the NO:Hb ratio increased by about 30% across the pulmonary vascular bed at low altitude (*P* < 0.001); similarly for both FeNO:Hb and SNO:Hb (Figure 1B). At high altitude, the NO:Hb ratio in arterial blood was significantly lower compared to venous blood (*P* < 0.01); similarly for FeNO:Hb and SNO:Hb. Venous NO:Hb, FeNO:Hb, and SNO:Hb increased 2-fold at high altitude compared to low altitude, and ascent (and hypobaric hypoxia) was associated with reversal of the gradient in erythrocyte NO:Hb ratios across the pulmonary bed.

Erythrocyte NO content and NO bound to hemoglobin as S-nitrosothiol does not differ between individuals susceptible and individuals resistant to HAPE. The development of high pulmonary artery pressure in subjects developing HAPE is unlikely to be caused by decreased hemoglobin transnitrosylation. However, we found changes in erythrocyte NO gradients across the pulmonary circulation with NO-loading at low and NO-offloading at high altitude in the lung. This suggests that increased peripheral production and loading of erythrocytes with NO may be a mechanism counterbalancing hypoxic pulmonary vasoconstriction.^3^ We studied a relatively small number of subjects because it was difficult to recruit subjects willing to re-expose themselves to high altitude after a previous episode of HAPE. However, when compared to other small studies, the numbers were sufficient for comparative analysis of erythrocyte NO content changes at high altitude. The NO:Hb ratios in our study were lower than those reported previously,^4^ yet when comparing values at low and high altitudes, the observed changes remain valid. The analyses were not adjusted for multiple comparisons, and the findings have to be interpreted with caution.

In conclusion, the development of excessively high pulmonary artery pressure in subjects susceptible to pulmonary edema at high altitude was not associated with decreased hemoglobin transnitrosylation in this study. At high altitude, there seems to be a reversal of the erythrocyte NO gradient across the pulmonary vasculature suggesting peripheral erythrocyte NO loading as a potential mechanism counterbalancing hypoxic pulmonary vasoconstriction at altitude.
